# Stress-activated pathways mediate PFAS effects on human placental syncytiotrophoblast cells

**DOI:** 10.1093/toxsci/kfag060

**Published:** 2026-05-22

**Authors:** Keisuke Kozai, Kaela M Varberg

**Affiliations:** Fetal Health Center, Children’s Mercy, Kansas City, MO 64108, United States; Fetal Health Center, Children’s Mercy, Kansas City, MO 64108, United States; Department of Pediatrics, University of Missouri Kansas City, Kansas City, MO 64108, United States

**Keywords:** PFAS mixtures, syncytiotrophoblast, JNK signaling, apoptosis, placenta

## Abstract

Per- and polyfluoroalkyl substances (PFAS) are persistent environmental pollutants associated with placenta-mediated pregnancy complications, including preeclampsia, fetal growth restriction, and preterm birth. The syncytiotrophoblast (STB), which forms the placental barrier at the maternal–fetal interface and is directly exposed to maternal blood, is a primary site of PFAS exposure. Although PFAS induce STB apoptosis, the upstream stress-signaling pathways involved remain poorly defined. Here, we investigated stress-responsive signaling mechanisms mediating PFAS-induced STB cell death. STB differentiated from human trophoblast stem cells were exposed to vehicle or an environmentally relevant mixture of 5 PFAS (perfluorooctanoic acid, perfluorooctanesulfonic acid, perfluorohexane sulfonate, perfluorononanoic acid, and perfluorodecanoic acid; 0.0138 to 34.5 µM) for 3 or 6 h. Cytotoxicity, apoptosis, mitochondrial membrane potential, and stress-signaling pathway activation were assessed by lactate dehydrogenase release, immunoblotting, JC-10 assay, and reverse transcription-quantitative PCR. PFAS mixtures did not induce cytotoxicity at 3 h but significantly increased cytotoxicity at 6 h at 34.5 µM, coinciding with the induction of cleaved caspase-3, cleaved poly(ADP-ribose) polymerase, and NOXA. The pan-caspase inhibitor z-VAD-FMK prevented cytotoxicity, indicating caspase-dependent apoptosis. PFAS exposure reduced mitochondrial membrane potential and activated the integrated stress response (ISR), as evidenced by eukaryotic initiation factor 2α phosphorylation, activating transcription factor 4 (ATF4) induction, and increased ATF4 target gene expression. In parallel, c-Jun N-terminal kinase (JNK) signaling was activated, as evidenced by JNK phosphorylation and induction of immediate-early genes (*JUN*, *FOS*, *EGR1*). Pharmacologic inhibition of the ISR modestly attenuated PFAS-induced cytotoxicity, whereas pharmacologic inhibition of JNK rescued cytotoxicity and apoptotic signaling. Together, these findings identify JNK-driven stress signaling as the dominant mediator of PFAS-induced STB apoptosis, with a secondary contribution from the ISR.

Per- and polyfluoroalkyl substances (PFAS) are persistent environmental contaminants of public health concern and are widely detected in human blood, including that of pregnant individuals (Buck et al. 2011; Sunderland et al. 2019; Cousins et al. 2020; Padula et al. 2023). Epidemiologic studies have associated PFAS exposure with adverse offspring outcomes spanning metabolic and neurodevelopmental domains, including altered adiposity/cardiometabolic markers (Manzano-Salgado et al. 2017; Papadopoulou et al. 2021; Zhang et al. 2023; Starling et al. 2024) and neurodevelopmental measures (Forns et al. 2020; Gao et al. 2023; Liu et al. 2024; Ames et al. 2025). Emerging evidence also links prenatal PFAS exposure to differences in offspring blood pressure trajectories (Li et al. 2025), suggesting potential impacts on early-life cardiovascular risk. In addition, prenatal PFAS exposure has been associated with placenta-mediated pregnancy complications, including fetal growth restriction (Wikstrom et al. 2020; Gui et al. 2022; Padula et al. 2023), preterm birth (Sagiv et al. 2018; Gui et al. 2022), and preeclampsia (Bommarito et al. 2021; Cathey et al. 2026). Despite these associations, the placental cell types and stress-responsive signaling pathways through which PFAS exposure disrupts placental function remain poorly defined.

The placenta is a transient organ that develops during pregnancy and performs critical functions required to establish and maintain a healthy pregnancy, including endocrine hormone production, regulated nutrient and gas exchange, and immunological barrier functions (Soares et al. 2018; Knofler et al. 2019). The syncytiotrophoblast (STB) forms the primary maternal–fetal interface and serves as the transport-regulating barrier of the placenta (Sibley 2009; Gaccioli et al. 2013). Because the STB is in direct contact with maternal blood, it represents a likely first site of interaction and uptake of xenobiotics within the placenta (Gaccioli et al. 2013; Walker et al. 2017), including PFAS that are detectable in placental tissue (Bangma et al. 2020; Hall et al. 2022; Perez et al. 2025b). Excessive STB stress and apoptosis are key pathological features of placenta-mediated pregnancy complications, including fetal growth restriction and preeclampsia (Sharp et al. 2010; Carrasco-Wong et al. 2021; Redman et al. 2022). Despite the central role of STB stress and apoptosis in these disorders, the upstream cellular pathways that precipitate this pathological response are not well characterized.

A growing body of evidence suggests that the placenta is an important target tissue for PFAS exposure (Blake and Fenton 2020). In mice, gestational exposure to perfluorooctanoic acid (PFOA), or its replacement compound GenX, results in adverse embryo–placenta outcomes, including reduced embryo weight and placental abnormalities localized to the labyrinth zone containing STB cells (Soares et al. 2018; Blake et al. 2020). In human models, perfluorooctanesulfonic acid (PFOS) induces caspase-dependent apoptosis in STB cells differentiated from primary cytotrophoblasts (Zhang et al. 2015). However, the upstream molecular mechanisms by which PFAS promote placental dysfunction, including STB apoptosis, remain incompletely understood, particularly at the level of early cellular stress responses (Blake and Fenton 2020).

Mitochondria are cellular organelles that function not only as cellular powerhouses but also as central hubs for sensing and integrating diverse cellular stress signals (Chandel 2014; Raimundo 2014). Mitochondrial dysfunction, often reflected by the loss of mitochondrial membrane potential, can engage key stress-response pathways (Soberanes et al. 2009; Ansari et al. 2020; Fessler et al. 2020; Fu et al. 2023). In addition, mitochondrial dysfunction is an early event in the intrinsic apoptotic pathway (Sanchez-Vera et al. 2026). PFAS have been reported to induce mitochondrial dysfunction, including decreased mitochondrial membrane potential, in the human choriocarcinoma trophoblast cell line JEG-3 (Blake et al. 2022; Zhao et al. 2022). Because mitochondria serve as central hubs for sensing and integrating cellular stress, PFAS-induced mitochondrial dysfunction may have important downstream consequences for stress-responsive signaling pathways.

The integrated stress response (ISR) is a conserved cellular adaptive pathway (Ryoo 2024). When cells encounter diverse stresses (e.g. xenobiotic stress, mitochondrial stress), eukaryotic initiation factor 2α (eIF2α) is phosphorylated by distinct stress-responsive kinases. Phosphorylation of eIF2α suppresses global translation while enabling preferential translation of select stress-responsive mRNAs. Among these, activating transcription factor 4 (ATF4) is a key ISR effector (Neill and Masson 2023). Under mild or transient stress, ATF4 primarily promotes adaptive programs, including amino acid metabolism and redox homeostasis. However, under severe or prolonged stress, sustained ISR signaling can shift toward prodeath outputs, including induction of proapoptotic genes such as *PMAIP1*, which encodes the BH3-only protein NOXA (Arai et al. 2020; Neill and Masson 2023; Zhang et al. 2026). PFOA has been reported to engage the eIF2α-ATF4 axis in the human trophoblast cell line HTR-8/SVneo (Du et al. 2022). In addition to ISR activation, PFAS exposure has been implicated in engagement of other stress-responsive signaling pathways relevant to trophoblast survival (Du et al. 2022; Zhao et al. 2022).

One such stress-responsive pathway is c-Jun N-terminal kinase (JNK), a stress-activated mitogen-activated protein kinase (MAPK) that responds to diverse stimuli, including oxidative stress and ultraviolet radiation (Leppa and Bohmann 1999). Upon activation via phosphorylation, JNK phosphorylates its target proteins, including the activator protein-1 (AP-1) transcription factors (e.g. c-Jun, ATF2) (Whitmarsh and Davis 1996; Schaeffer and Weber 1999; Davis 2000). Increased AP-1 activity results in the induction of immediate-early genes, including *JUN*, *FOS*, and *EGR1*, which are commonly used as transcriptional readouts of stress-activated MAPK signaling (Ventura et al. 2003; Ito et al. 2025). The JNK signaling pathway can promote either cell survival or apoptosis in a context- and stimulus-dependent manner (Liu and Lin 2005). PFOS has been reported to activate JNK in the human choriocarcinoma trophoblast cell line JEG-3 (Zhao et al. 2022). However, whether JNK and related stress-responsive pathways are engaged in primary human STB models remains unclear.

In this study, we used human trophoblast stem cell-derived STB (Okae et al. 2018) as a model to investigate molecular mechanisms by which PFAS disrupt STB homeostasis. PFAS comprise thousands of structurally related compounds, but PFOA, PFOS, perfluorohexane sulfonate (PFHxS), perfluorononanoic acid (PFNA), and perfluorodecanoic acid (PFDA) are among the PFAS most consistently quantified in pregnancy cohorts and frequently detected in maternal biospecimens (Padula et al. 2023). These 5 PFAS compounds are also highly prevalent in placental tissue (Hall et al. 2022; Perez et al. 2025a). To model real-world exposure, we formulated PFAS mixtures containing these 5 compounds at ratios based on their geometric mean concentrations in maternal blood from the Environmental Influences on Child Health Outcomes (ECHO) Program (Padula et al. 2023). We tested the hypothesis that exposure to PFAS mixtures induces mitochondrial dysfunction and activates the ISR and JNK stress-signaling pathways, thereby promoting STB apoptosis and cytotoxicity. By integrating environmentally informed exposure modeling with human STB, this study provides new mechanistic insight into PFAS-induced placental stress and injury.

## Materials and methods

### Chemicals

PFOA (98.0% purity; 2121-3-18, CAS: 335-67-1), PFOS (95.0% purity; 6164-3-08, CAS: 1763-23-1), PFNA (97.0% purity; 2121-3-20, CAS: 375-95-1), and PFDA (98.0% purity; 2121-3-24, CAS: 335-76-2) were obtained from Synquest Laboratories (Alachua, FL). Potassium PFHxS (099754, CAS: 3871-99-6) was obtained from Matrix Scientific (Columbia, South Carolina). Each PFAS compound was dissolved in potassium hydroxide 1.0 N in methanol (LC19540, LabChem, Zelienople, Pennsylvania). Z-VAD(OMe)-FMK (HY-16658), staurosporine (HY-15141), and JNK-IN-8 (HY-13319) were obtained from MedChemExpress (Monmouse Junction, New Jersey). Trans-ISRIB (75845) was obtained from Cell Signaling Technology (Danvers, Massachusetts).

### Trophoblast stem cell culture and PFAS exposure

A cytotrophoblast-derived CT29 (XY) trophoblast stem cell line was used in this study. Human trophoblast stem cells were maintained in the stem state or differentiated into STB cells as described previously (Okae et al. 2018). Human trophoblast stem cells were cultured in 100 mm cell culture dishes (172931, Thermo Fisher Scientific, Waltham, Massachusetts) coated with 5 µg/ml of collagen IV (C5533, Sigma-Aldrich, St. Louis, Missouri) in Complete Trophoblast Stem Cell Medium (Dulbecco’s Modified Eagle Medium/F12 [DMEM/F12, 11320033, Thermo Fisher Scientific], 50 U/ml penicillin, 50 µg/ml streptomycin [15140122, Thermo Fisher Scientific], 100 µM 2-Mercaptoethanol [M3148, Sigma-Aldrich], 0.2% [vol/vol] fetal bovine serum [16141079, Thermo Fisher Scientific], 0.3% bovine serum albumin [BSA, BP9704100, Thermo Fisher Scientific], 1% (vol/vol) Insulin–Transferrin–Selenium–Ethanolamine solution [ITS-X, 51500056, Thermo Fisher Scientific], 1.5 µg/ml L-ascorbic acid [A8960, Sigma-Aldrich], 50 ng/ml epidermal growth factor [EGF, E9644, Sigma-Aldrich], 2 µM CHIR99021 [04-0004, Reprocell, Beltsville, Maryland], 0.5 µM A83-01 [04-0014, Reprocell], 1 µM SB431542 [04-0010, Reprocell], 0.8 mM valproic acid [P4543, Sigma-Aldrich], and 5 µM Y27632 [04-0012, Reprocell]). For STB differentiation, trophoblast stem cells were plated into non-tissue culture-treated petri dishes (351007, Corning Inc., Corning, NY) at a density of 300,000 cells per dish (100,000 cells/ml) and cultured in ST three-dimensional (ST3D) Medium (DMEM/F12 [11320033, Thermo Fisher Scientific], 50 U/ml penicillin, 50 µg/ml streptomycin [15140122, Thermo Fisher Scientific], 100 µM 2-Mercaptoethanol [M3148, Sigma-Aldrich], 0.3% BSA [BP9704100, Thermo Fisher Scientific], 1% ITS-X [51500056, Thermo Fisher Scientific], 2.5 µM Y27632 [04-0012, Reprocell], 4% KnockOut Serum Replacement [KSR, 10828028, Thermo Fisher Scientific], 2 µM forskolin [F6886, Sigma-Aldrich], and 50 ng/ml EGF [E9644, Sigma-Aldrich]).

PFAS exposure was initiated at 48 h following STB differentiation. On the day of PFAS exposure, STB cells were centrifuged at 380×*g* for 2 min, supernatants were removed, and STB cells were resuspended in fresh ST3D Medium without KSR/BSA and exposed to vehicle (0.35% MeOH) or PFAS mixtures (Table 1; 0.0138 to 34.5 µM total concentration).

**Table 1.** Maternal PFAS serum concentrations in the ECHO study and PFAS concentrations used in this study.

		Maternal serum (ECHO)					
PFAS (full name)	Abbreviation	Geometric mean (µM)	Concentrations used in this study (µM)				
Perfluorooctanoic acid	PFOA	0.0028	0.0028	0.028	0.28	2.8	7
Perfluorooctane sulfonate	PFOS	0.0077	0.0077	0.077	0.77	7.7	19.25
Perfluorohexane sulfonate	PFHxS	0.0023	0.0023	0.023	0.23	2.3	5.75
Perfluorononanoic acid	PFNA	0.0008	0.0008	0.008	0.08	0.8	2
Perfluorodecanoic acid	PFDA	0.0002	0.0002	0.002	0.02	0.2	0.5
**Total concentrations** ^#^		**0.0138**	**0.0138**	**0.138**	**1.38**	**13.8**	**34.5**

# Bold text indicates total mixture concentrations.

**Table 2.** RT-qPCR primers.

Gene	Orientation	Sequence (5′→3′)	Accession no.	Amplicon size (bp)
*ASNS*	Forward	CTGTGAAGAACAACCTCAGGATC	NM_133436.3	124
	Reverse	AACAGAGTGGCAGCAACCAAGC		
*PPP1R15A*	Foward	TCCGACTGCAAAGGCGGCTCA	NM_014330.5	120
	Reverse	CAGCCAGGAAATGGACAGTGAC		
*DDIT3*	Forward	GGAGCTGGAAGCCTGGTATG	NM_001195053.1	132
	Reverse	AGAGAAGCAGGGTCAAGAGTGG		
*CHAC1*	Forward	GTGGTGACGCTCCTTGAAGATC	NM_024111.6	144
	Reverse	GAAGGTGACCTCCTTGGTATCG		
*TRIB3*	Forward	GCTTTGTCTTCGCTGACCGTGA	NM_021158.5	136
	Reverse	CTGAGTATCTCAGGTCCCACGT		
*ATF3*	Forward	TCGGGGTGTCCATCACAA	NM_001674.4	120
	Reverse	CCGTCTTCTCCTTCTTCTTGTTTC		
*JUN*	Forward	CCTTGAAAGCTCAGAACTCGGAG	NM_002228.4	124
	Reverse	TGCTGCGTTAGCATGAGTTGGC		
*FOS*	Forward	GCCTCTCTTACTACCACTCACC	NM_005252.4	126
	Reverse	AGATGGCAGTGACCGTGGGAAT		
*EGR1*	Forward	AGCAGCACCTTCAACCCTCAGG	NM_001964.3	133
	Reverse	GAGTGGTTTGGCTGGGGTAACT		
*PMAIP1*	Forward	CTGGAAGTCGAGTGTGCTACTC	NM_001382616.1	139
	Reverse	TGAAGGAGTCCCCTCATGCAAG		
*PPIB*	Forward	GACTTCACCAGGGGAGATGG	NM_000942.5	126
	Reverse	GGTGTCTTTGCCTGCGTTG		

### Cytotoxicity

To assess the cytotoxicity of PFAS mixtures on STB, lactate dehydrogenase (LDH) released into the supernatants was quantified using the Cytotoxicity LDH Assay Kit-WST (CK12, Dojindo, Rockville, Maryland), according to the manufacturer’s instructions. Absorbance was measured at 490 nm using a CLARIOstar PLUS plate reader (BMG LABTECH, Cary, North Carolina). The low control represented LDH activity released from untreated cells, and the high control represented total cellular LDH activity. For the high control, cells were treated with the lysis buffer provided with the kit for 30 min. LDH release (% of total) was calculated as:


$$\mathrm{LDH}\;\mathrm{release}\;(\%)=\frac{\mathrm{OD}490\;(\mathrm{test}\;\mathrm{substance})-\mathrm{OD}490\;(\mathrm{low}\;\mathrm{control})}{\mathrm{OD}490\;(\mathrm{high}\;\mathrm{control})-\mathrm{OD}490\;(\mathrm{low}\;\mathrm{control})}\times 100$$

After calculating LDH release (% of total) as described above, values were normalized to the vehicle control by subtracting the mean LDH release of the vehicle-treated wells from each sample. Thus, the vehicle control was set to 0% cytotoxicity.

### Simple Western analysis

Cell lysates were prepared using the radioimmunoprecipitation assay lysis buffer system (sc-24948A, Santa Cruz Biotechnology, Dallas, Texas). Protein concentrations were determined using a Pierce BCA Protein Assay Kit (23227, Thermo Fisher Scientific) according to the manufacturer’s instructions. Protein expression was analyzed using the Jess Simple Western system (ProteinSimple, San Jose, California). Primary antibodies to cleaved caspase-3 (CC3; 1:50, 9662), cleaved poly(ADP-ribose) polymerase (cPARP) (1:250, 9541), phospho-eIF2α (Ser51) (1:50, 9721), eIF2α (1:250, 5324), phospho-JNK (Thr183/Tyr185) (1:50, 4668), JNK (1:50, 9252), and glyceraldehyde 3-phosphate dehydrogenase (1:250, 2118) were purchased from Cell Signaling Technology (Danvers, Massachusetts). A primary antibody to NOXA (1:50, NBP3-07012SS) was purchased from Novus Biologicals (Centennial, Colorado).

### Mitochondrial membrane potential assay

To assess the effects of PFAS mixtures on mitochondrial membrane potential, we measured mitochondrial membrane potential using the JC-10 Mitochondrial Membrane Potential Assay Kit (ab112134, Abcam, Cambridge, United Kingdom) according to the manufacturer’s instructions. Fluorescence intensities were measured at excitation/emission = 488/535 nm and 540/590 nm for ratio analysis using a CLARIOstar PLUS plate reader (BMG LABTECH). Mitochondrial membrane potential was expressed as the 590/535 fluorescence ratio. Staurosporine (1 µM) was used as a positive control.

### Transcript analysis

Total RNA was purified from cells using the RNeasy Plus Mini Kit (74136, QIAGEN, Germantown, MD) according to the manufacturer’s instructions. Total RNA (1 µg) was reverse transcribed using a High-Capacity cDNA Reverse Transcription Kit (4368813, Thermo Fisher Scientific). Complementary DNA was diluted 1:10 and subjected to reverse transcription-quantitative PCR using Power SYBR Green PCR Master Mix (4367659, Thermo Fisher Scientific) and the primers provided in Table 2. QuantStudio 12K Flex Real-Time PCR System (Applied Biosystems) was used for amplification and fluorescence detection. PCR was performed under the following conditions: 50 °C for 2 min and 95 °C for 10 min, followed by 40 cycles of 95 °C for 15 s and 60 °C for 1 min. *PPIB* was used as a reference transcript.

### Statistical analysis

Statistical analyses were performed with GraphPad Prism 10 software. Following one-way ANOVA, Dunnett’s or Tukey’s multiple comparison test was performed where appropriate. Results were deemed statistically significant when *P* < 0.05.

## Results

### PFAS mixtures induce caspase-dependent apoptosis and cytotoxicity in STB cells

**Fig. 1.**
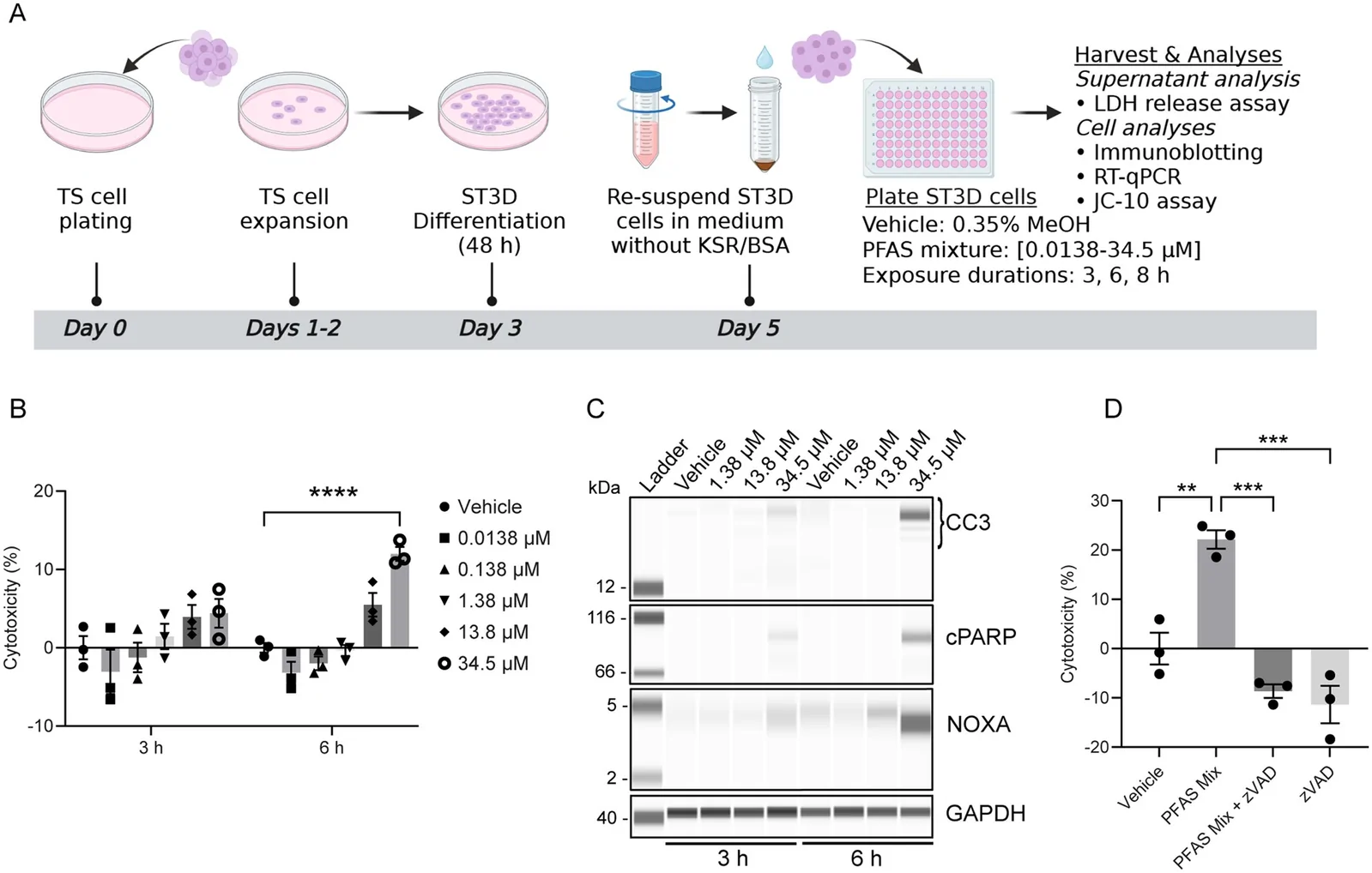
PFAS mixture exposure induces cytotoxicity in STB cells. (A) A simplified schematic outlining the study design. Created in BioRender. Kozai, K. (2026) https://BioRender.com/7ryy9qa (B) Cytotoxicity was evaluated using an LDH assay after 3 or 6 h of exposure to vehicle (0.35% MeOH) or PFAS mixtures (0.0138 to 34.5 µM). Bars represent mean ± SEM (*n* = 3/group). Asterisks denote statistical significance compared with vehicle (****P < 0.0001), as determined by Dunnett’s test. (C) Simple Western analysis of cleaved caspase-3 (CC3), cleaved poly(ADP-ribose) polymerase (cPARP), and NOXA protein in STB cells exposed to vehicle (0.35% MeOH) or PFAS mixtures (1.38 to 34.5 µM) for 3 or 6 h. GAPDH was used as a loading control. (D) Cytotoxicity was evaluated using an LDH assay after 8 h of exposure to vehicle (0.35% MeOH) or PFAS mixtures (PFAS Mix; 34.5 µM), with or without pretreatment with z-VAD(OMe)-FMK (zVAD; 50 µM, 1 h). Bars represent mean ± SEM (*n* = 3/group). Asterisks denote statistical significance (**P < 0.01; ***P < 0.001), as determined by Tukey’s multiple comparisons test.

To evaluate the effect of PFAS mixtures on cytotoxicity and apoptosis, STB cells were exposed to either vehicle control (0.35% methanol) or PFAS mixtures (Table 1; 0.0138 to 34.5 µM total concentration) for 3 or 6 h (Fig. 1A). At 3 h, PFAS mixtures did not induce detectable cytotoxicity. By contrast, 34.5 µM PFAS mixtures significantly increased cytotoxicity at 6 h (Fig. 1B). Consistent with these observations, apoptotic markers (CC3, cPARP, and NOXA) were detectable as early as 3 h and further increased at 6 h following exposure to 34.5 µM PFAS mixtures (Fig. 1C). We next examined whether PFAS mixture-induced cytotoxicity was mediated by apoptosis. STB cells were pretreated with the pan-caspase inhibitor Z-VAD(OMe)-FMK (50 µM) for 1 h and then exposed to 34.5 µM PFAS mixtures for 8 h. Pretreatment with Z-VAD(OMe)-FMK significantly inhibited PFAS mixture-induced cytotoxicity (Fig. 1D). Together, these results indicate that PFAS mixture-induced cytotoxicity in STB cells is mediated by caspase-dependent apoptotic cell death.

### PFAS mixtures trigger mitochondrial dysfunction in STB cells

**Fig. 2.**
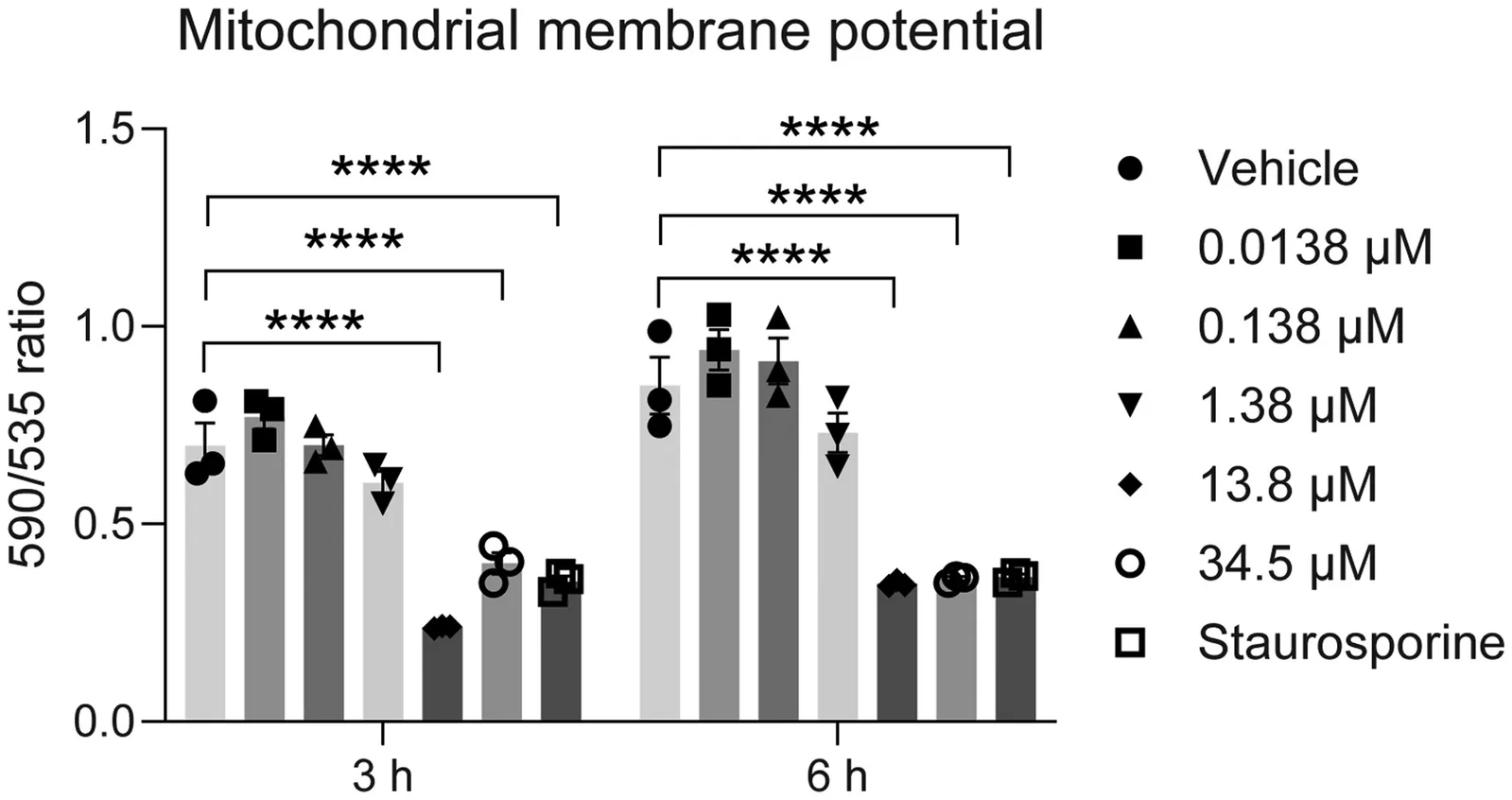
PFAS mixture exposure reduces mitochondrial membrane potential in STB cells. Mitochondrial membrane potential was measured using a JC-10 assay after 3 or 6 h of exposure to vehicle (0.35% MeOH) or PFAS mixtures (0.0138 to 34.5 µM). Bars represent mean ± SEM (*n* = 3/group). Asterisks denote statistical significance compared with vehicle (****P < 0.0001), as determined by Dunnett’s test. Staurosporine (1 µM) was used as a positive control.

To identify upstream events associated with apoptosis, we examined whether PFAS mixtures induce mitochondrial dysfunction. We measured mitochondrial membrane potential as an indicator of mitochondrial dysfunction following PFAS exposure. At 3 and 6 h, both 13.8 and 34.5 µM PFAS mixtures significantly decreased mitochondrial membrane potential (Fig. 2). These results indicate that PFAS mixtures induce mitochondrial dysfunction in STB cells.

### PFAS mixtures activate the ISR in STB cells

**Fig. 3.**
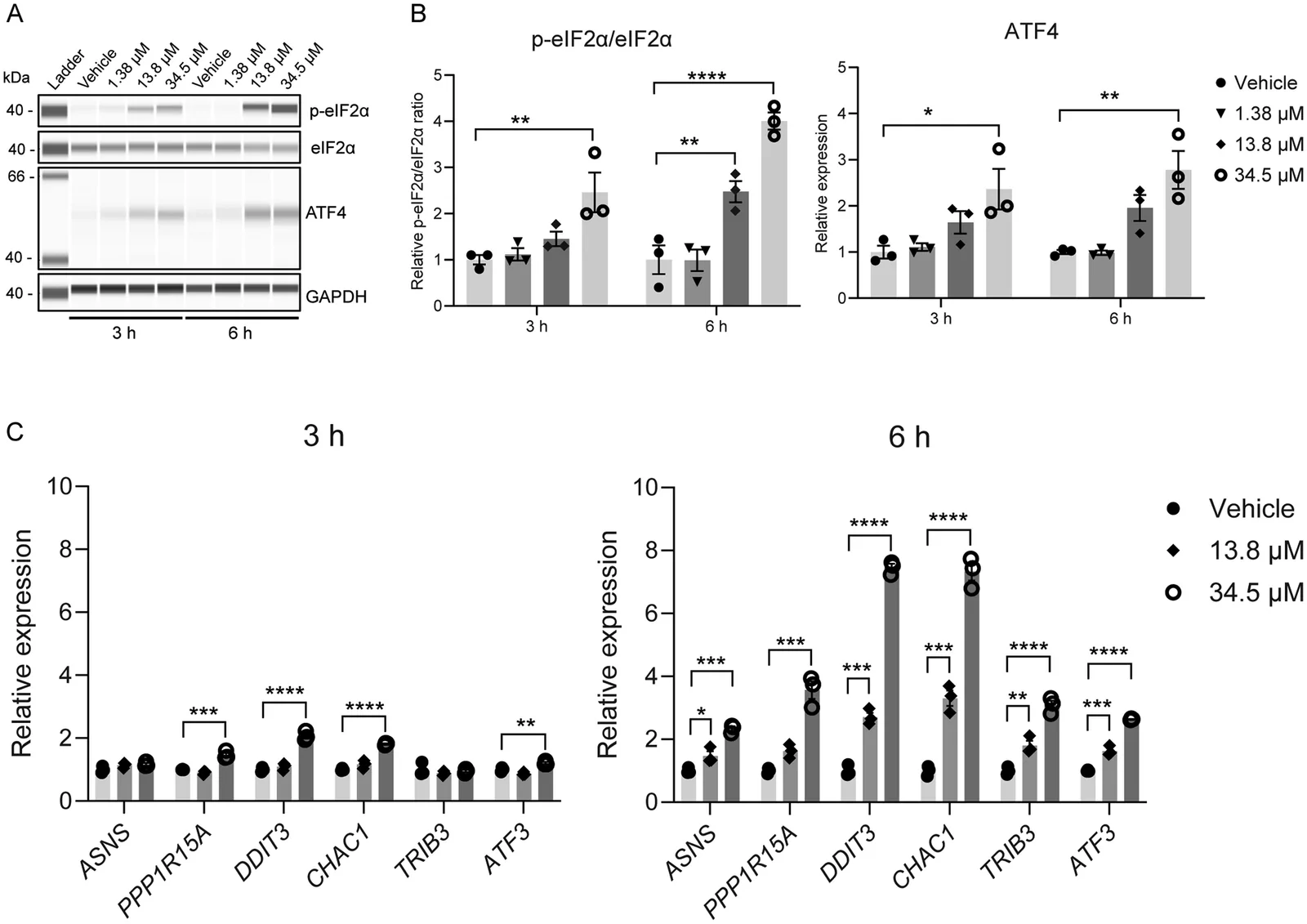
PFAS mixtures activate the ISR in STB cells. (A) Simple Western analysis of phosphorylated (p)-eIF2α, eIF2α, and ATF4 protein in STB cells exposed to vehicle (0.35% MeOH) or PFAS mixtures (1.38 to 34.5 µM) for 3 or 6 h. GAPDH was used as a loading control. (B) p-eIF2α/eIF2α ratio and ATF4 protein expression relative to vehicle control. Bars represent mean ± SEM (*n* = 3/group). Asterisks denote statistical significance compared with vehicle (*P < 0.05; **P < 0.01; ****P < 0.0001), as determined by Dunnett’s test. (C) RT-qPCR measurements of *ASNS*, *PPP1R15A*, *DDIT3*, *CHAC1*, *TRIB3*, and *ATF3* transcripts in STB cells exposed to vehicle (0.35% MeOH) or PFAS mixtures (13.8 and 34.5 µM) for 3 or 6 h. Bars represent mean ± SEM (*n* = 3/group). Asterisks denote statistical significance compared with vehicle (*P < 0.05; **P < 0.01; ***P < 0.001; ****P < 0.0001), as determined by Dunnett’s test.

Given the mitochondrial dysfunction observed after PFAS exposure, we next assessed whether PFAS mixtures activate the ISR in STB cells, as indicated by eIF2α phosphorylation and ATF4 induction (Neill and Masson 2023). At 3 h, 34.5 µM PFAS mixtures significantly increased the phosphorylated eIF2α (p-eIF2α)/eIF2α ratio, upregulated ATF4 protein, and induced ATF4 target transcripts including *PPP1R15A*, *DDIT3*, *CHAC1*, and *ATF3* (Fig. 3). At 6 h, 13.8 µM PFAS mixtures significantly increased the p-eIF2α/eIF2α ratio and induced a subset of ATF4 target transcripts, whereas 34.5 µM PFAS mixtures produced more robust ISR activation, with increased ATF4 protein and broader induction of ATF4 target transcripts including *ASNS*, *PPP1R15A*, *DDIT3*, *CHAC1*, *TRIB3*, and *ATF3* (Fig. 3). These results indicate that PFAS mixtures activated the ISR in STB cells in a dose- and time-dependent manner.

### PFAS mixtures activate the JNK signaling pathway in STB cells

**Fig. 4.**
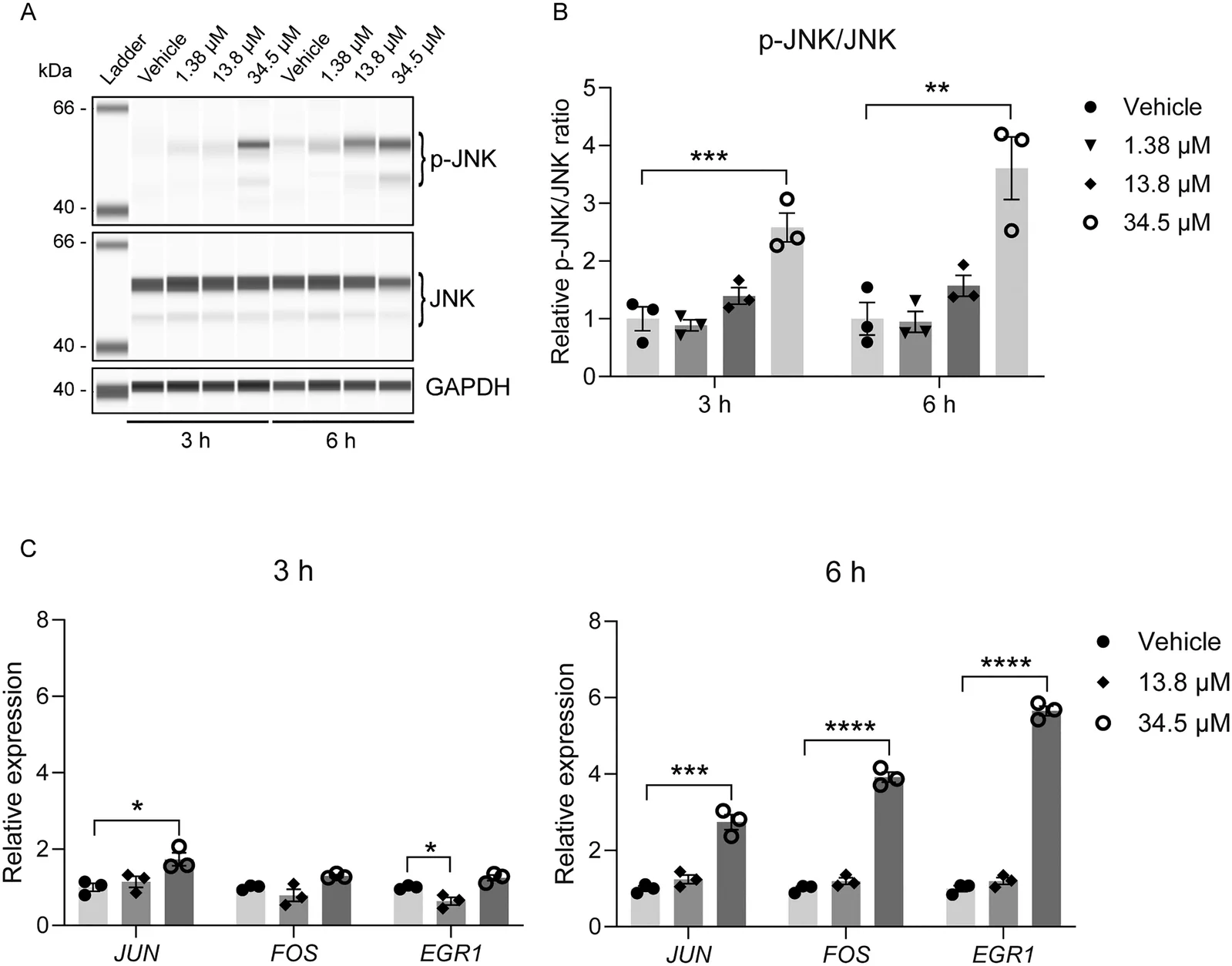
PFAS mixtures activate JNK signaling. (A) Simple Western analysis of phosphorylated (p)-JNK and JNK protein in STB cells exposed to vehicle (0.35% MeOH) or PFAS mixtures (1.38 to 34.5 µM) for 3 or 6 h. GAPDH was used as a loading control. (B) p-JNK/JNK ratio relative to vehicle control. Bars represent mean ± SEM (*n* = 3/group). Asterisks denote statistical significance compared with vehicle (**P < 0.01; ***P < 0.001), as determined by Dunnett’s test. (C) RT-qPCR measurements of *JUN*, *FOS*, and *EGR1* transcripts in STB cells exposed to vehicle (0.35% MeOH) or PFAS mixtures (13.8 and 34.5 µM) for 3 or 6 h. Bars represent mean ± SEM (*n* = 3/group). Asterisks denote statistical significance compared with vehicle (*P < 0.05; ***P < 0.001; ****P < 0.0001), as determined by Dunnett’s test.

Because PFAS mixtures activated the ISR, we next assessed whether PFAS mixtures also activate the JNK signaling pathway in STB cells. At 3 and 6 h, 34.5 µM PFAS mixtures significantly increased the phosphorylated JNK/JNK ratio (Fig. 4A and B). Expression of immediate-early genes (*JUN*, *FOS*, and *EGR1*) showed variable induction at 13.8 and 34.5 µM PFAS mixture exposures after 3 h; however, all 3 transcripts were upregulated by 34.5 µM PFAS mixtures at 6 h (Fig. 4C). Collectively, these results indicate that PFAS mixtures activate JNK signaling in STB cells in a dose- and time-dependent manner.

### PFAS mixture-induced cytotoxicity is primarily mediated by JNK signaling

**Fig. 5.**
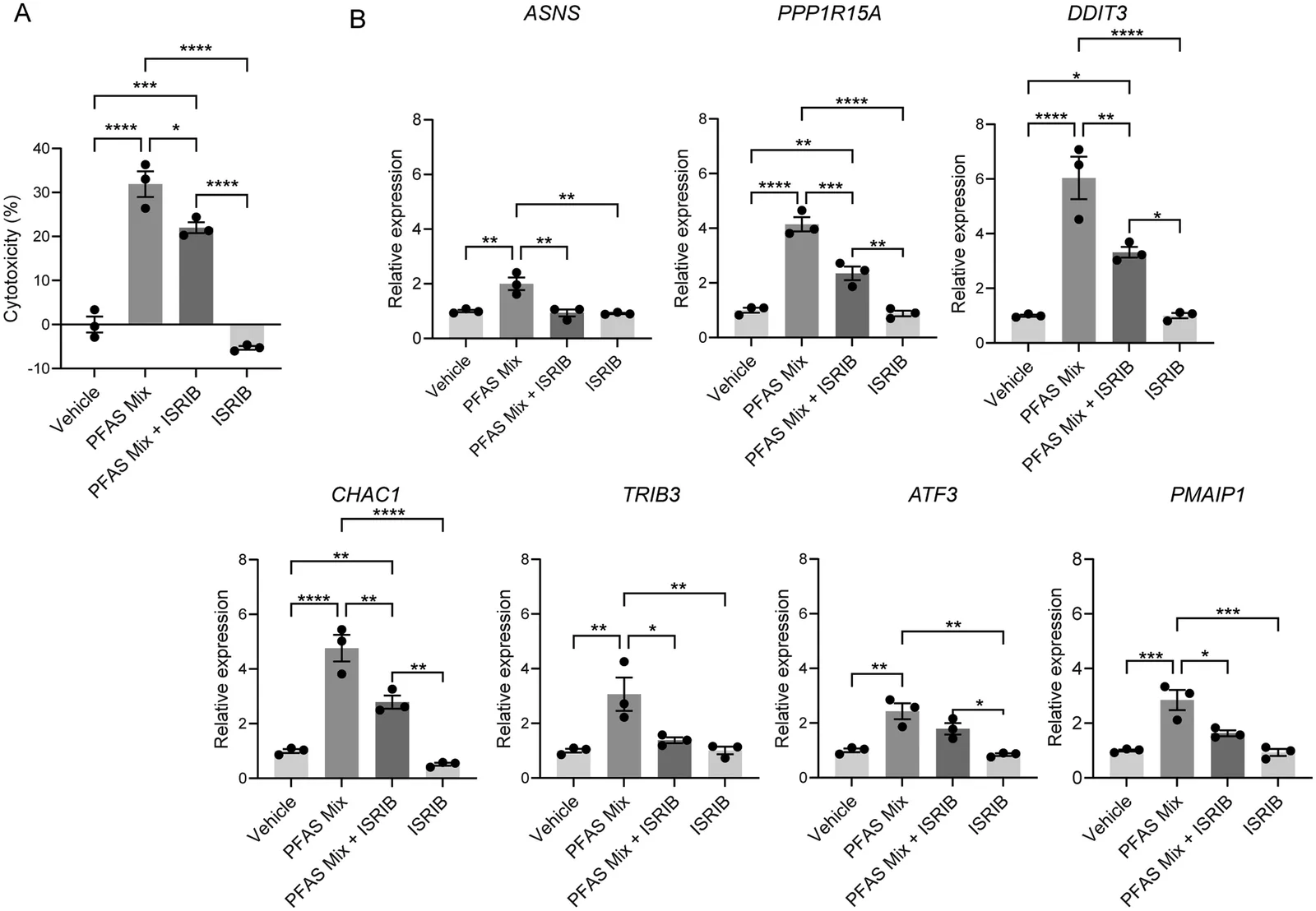
ISR inhibition partially rescued PFAS-induced cytotoxicity. (A) Cytotoxicity was evaluated using an LDH assay after 8 h of exposure to vehicle (0.35% MeOH) or PFAS mixtures (PFAS Mix; 34.5 µM), with or without pretreatment with ISRIB (250 nM, 1 h). Bars represent mean ± SEM (*n* = 3/group). Asterisks denote statistical significance (*P < 0.05; ***P < 0.001; ****P < 0.0001), as determined by Tukey’s multiple comparisons test. (B) RT-qPCR measurements of *ASNS*, *PPP1R15A*, *DDIT3*, *CHAC1*, *TRIB3*, *ATF3*, and *PMAIP1* transcripts in STB cells exposed to vehicle (0.35% MeOH) or PFAS mixtures (PFAS Mix; 34.5 µM) for 8 h, with or without pretreatment with ISRIB (250 nM, 1 h). Bars represent mean ± SEM (*n* = 3/group). Asterisks denote statistical significance (*P < 0.05; **P < 0.01; ***P < 0.001; ****P < 0.0001), as determined by Tukey’s multiple comparisons test.

**Fig. 6.**
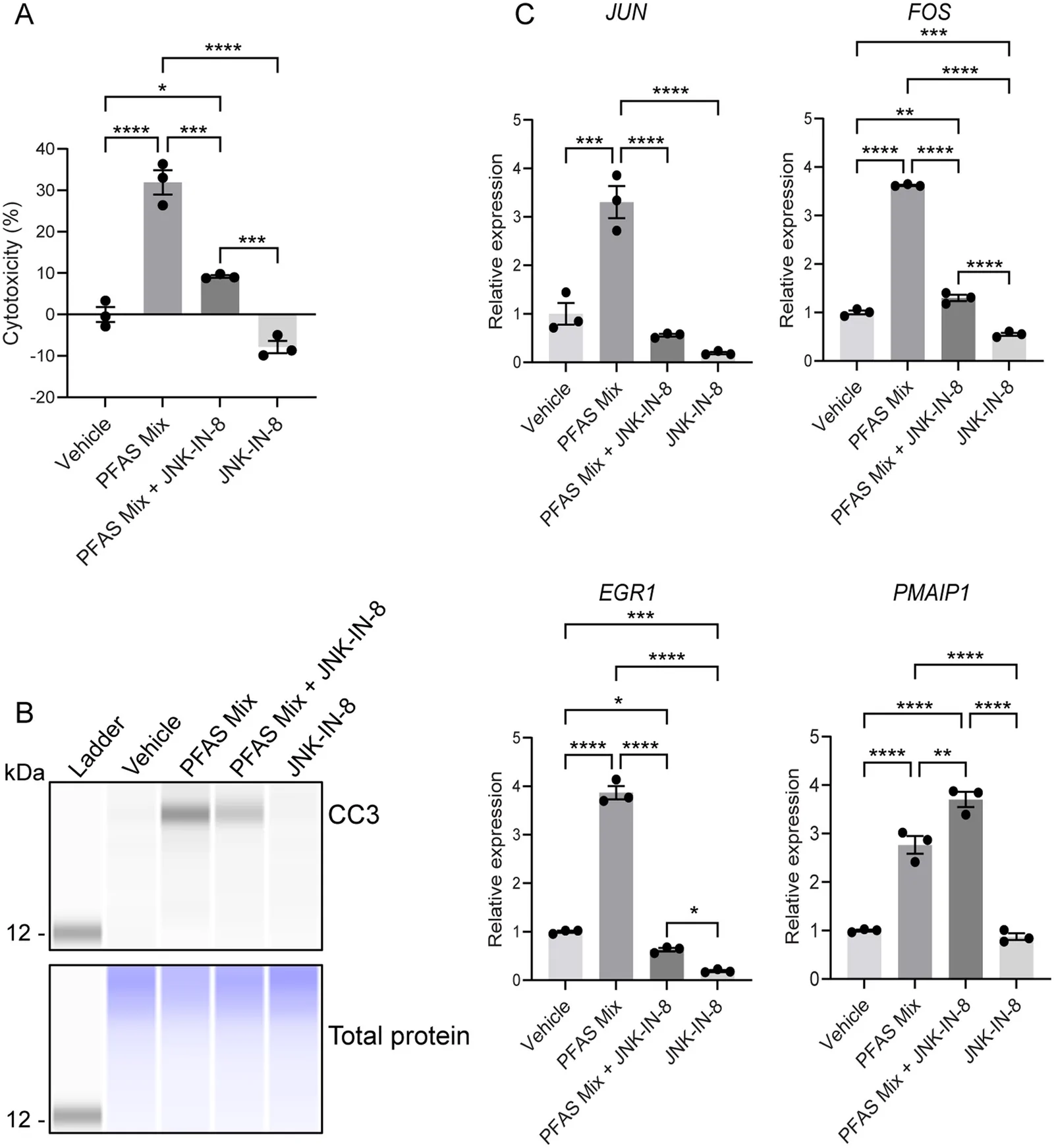
JNK inhibition rescued PFAS-induced cytotoxicity. (A) Cytotoxicity was evaluated using an LDH assay after 8 h of exposure to vehicle (0.35% MeOH) or PFAS mixtures (PFAS Mix; 34.5 µM), with or without pretreatment with JNK-IN-8 (1 µM, 1 h). Bars represent mean ± SEM (*n* = 3/group). Asterisks denote statistical significance (*P < 0.05; ***P < 0.001; ****P < 0.0001), as determined by Tukey’s multiple comparisons test. (B) Simple Western analysis of cleaved caspase-3 (CC3) in STB cells exposed to vehicle (0.35% MeOH) or PFAS mixtures (PFAS Mix; 34.5 µM) for 8 h, with or without pretreatment with JNK-IN-8 (1 µM, 1 h). Total protein was used as a loading control. (C) RT-qPCR measurements of *JUN*, *FOS*, *EGR1*, and *PMAIP1* transcripts in STB cells exposed to vehicle (0.35% MeOH) or PFAS mixtures (PFAS Mix; 34.5 µM) for 8 h, with or without pretreatment with JNK-IN-8 (1 µM, 1 h). Bars represent mean ± SEM (*n* = 3/group). Asterisks denote statistical significance (*P < 0.05; **P < 0.01; ***P < 0.001; ****P < 0.0001), as determined by Tukey’s multiple comparisons test.

Both the ISR and JNK signaling pathways have been implicated in apoptosis. We therefore examined whether the ISR and JNK pathways contribute to PFAS mixture-induced cytotoxicity. Pharmacological inhibition of the ISR with ISRIB (250 nM) modestly but significantly reduced 34.5 µM PFAS mixture-induced cytotoxicity (Fig. 5A). Consistent with these findings, ISRIB partially but significantly attenuated 34.5 µM PFAS mixture-induced expression of ATF4 target transcripts (*ASNS*, *PPP1R15A*, *DDIT3*, *CHAC1*, *TRIB3*, *ATF3*, *PMAIP1*; Fig. 5B). By contrast, pharmacological inhibition of JNK with JNK-IN-8 (1 µM) markedly and significantly reduced 34.5 µM PFAS mixture-induced cytotoxicity (Fig. 6A). JNK-IN-8 also decreased CC3 protein expression, which is induced by 34.5 µM PFAS mixture (Fig. 6B). JNK-IN-8 significantly suppressed induction of *JUN*, *FOS*, and *EGR1* transcripts by 34.5 µM PFAS mixtures (Fig. 6C). *PMAIP1*, which encodes NOXA, is a common downstream target of both the ISR and JNK signaling pathways. ISRIB, but not JNK-IN-8, significantly attenuated induction of *PMAIP1* transcripts by 34.5 µM PFAS mixtures (Figs 5B and 6C). Thus, although both pathways are engaged, JNK signaling plays a dominant role in PFAS mixture-induced cytotoxicity, whereas the ISR exerts a secondary effect through regulation of proapoptotic targets such as NOXA.

## Discussion

In this study, we define stress-responsive signaling pathways that mediate PFAS mixture-induced cytotoxicity in human STB cells. Using trophoblast stem cell-derived STB exposed to an environmentally informed mixture of 5 PFAS commonly detected in peripheral blood, we show that PFAS exposure induces mitochondrial dysfunction, activates multiple cellular stress pathways, and culminates in caspase-dependent apoptosis. Notably, our findings identify JNK signaling as the dominant mediator of PFAS-induced STB cell death, whereas activation of the ISR contributes more modestly. These results refine current understanding of PFAS placental toxicity by establishing a functional hierarchy among stress pathways engaged by PFAS mixtures.

PFAS have been demonstrated to induce apoptosis in various cell types, including STB cells (Zhang et al. 2015; Du et al. 2022; Han and Park 2023). Cleaved caspase-3, cPARP, and NOXA were detected earlier than overt cytotoxicity following PFAS exposure, suggesting that PFAS-induced STB cell death is mediated via apoptosis. Indeed, the pan-caspase inhibitor z-VAD-FMK inhibited PFAS-induced cytotoxicity. These results indicate that PFAS mixtures induce caspase-dependent apoptotic cell death in STB cells.

PFAS exposure has been implicated in mitochondrial dysfunction (Zhu et al. 2026). In this study, mitochondrial dysfunction emerged as an early cellular response to PFAS exposure and likely represents an upstream event linking PFAS exposure to downstream stress signaling and apoptosis. Reduction in mitochondrial membrane potential occurred at concentrations and time points before or alongside detection of apoptotic markers and preceding overt cytotoxicity, suggesting that mitochondrial dysfunction is an upstream event of PFAS-induced cytotoxicity in STB cells. The data also fit well with previous reports demonstrating that reduction in mitochondrial membrane potential is an early step of apoptosis (Zamzami et al. 1995a, 1995b, 1996). Mitochondrial dysfunction is known to engage both the ISR (Fessler et al. 2020; Guo et al. 2020; Fu et al. 2023) and JNK signaling (Dougherty et al. 2004; Ansari et al. 2020), providing a plausible mechanistic basis for the coordinated activation of the ISR and JNK observed here. These findings support a model in which PFAS-induced mitochondrial dysfunction initiates parallel stress signaling cascades that ultimately favor apoptosis. However, further mechanistic studies are needed to establish these relationships.

A central finding of this work is the prominent role of JNK signaling in mediating PFAS-induced apoptosis in STB cells. PFAS have been shown to activate JNK in several placental cell lines, including JEG-3 (Zhu et al. 2015; Sun et al. 2019; Zhao et al. 2022). In this study, PFAS mixtures activated JNK, as indicated by increased JNK phosphorylation and induction of canonical downstream target genes. PFOS has been demonstrated to induce apoptosis via JNK signaling in the human neuroblastoma cell line SH-SY5Y (Sun et al. 2019). Consistent with this, pharmacologic inhibition of JNK markedly attenuated PFAS-induced cytotoxicity and apoptotic signaling in STB cells, demonstrating that JNK activity is the dominant cytotoxic driver. JNK signaling can promote apoptosis through both transcription-dependent mechanisms, including induction of proapoptotic genes, and transcription-independent mechanisms involving phosphorylation of apoptotic regulators (Liu and Lin 2005; Dhanasekaran and Reddy 2008). Further research is needed to determine mechanisms by which JNK promotes apoptosis in response to PFAS mixtures in STB cells.

PFAS mixtures also activated the ISR, as evidenced by phosphorylation of eIF2α, induction of ATF4 protein, and increased expression of ATF4 target genes. Pharmacological inhibition of the ISR modestly but significantly reduced PFAS-induced cytotoxicity, supporting a contributory role for ISR signaling in shaping the cellular response to PFAS exposure. However, the comparatively limited protective effect of ISR inhibition relative to JNK blockade indicates that ISR activation alone is insufficient to drive extensive cell death. Instead, our findings support a model in which the ISR modulates apoptotic priming, at least in part, by inducing *PMAIP1* (NOXA) as described previously (Bajpai et al. 2020; Winter et al. 2022). The ISR possesses dual adaptive and prodeath functions, which are highly dependent on stress severity and duration (Altintas et al. 2026). Indeed, lower, noncytotoxic concentrations of PFAS mixtures activated the ISR without triggering overt cytotoxicity. It is possible that PFAS mixtures at lower concentrations engage the ISR without exceeding a certain threshold of activation required to shift from an adaptive response toward proapoptotic signaling. Whether ISR activation at lower PFAS concentrations promotes adaptive stress tolerance in STB cells will require further investigation.

The concurrent activation of ISR and JNK highlights the complexity of stress signaling networks in placental cells and underscores the importance of pathway hierarchy in determining toxicologic outcomes. Both pathways can promote either adaptive or prodeath responses depending on context (Liu and Lin 2005; Altintas et al. 2026). However, our results indicate that in STB cells exposed to PFAS mixtures, JNK signaling predominates in driving cytotoxicity. Given the essential role of STB in maintaining placental barrier and endocrine functions, excessive activation of JNK signaling may have disproportionate consequences for placental homeostasis. These findings suggest that stress intensity and pathway dominance, rather than pathway activation, are key determinants of PFAS-induced placental toxicity.

Major strengths of this study are the use of an environmentally informed PFAS mixture rather than single-compound exposures and a human trophoblast stem cell-derived STB model. Because human exposure to PFAS occurs as co-exposure to multiple PFAS, mixture-based effects should be considered, and interactions among PFAS may influence stress signaling dynamics and toxicologic outcomes (Rios-Bonilla et al. 2024). By modeling real-world exposure profiles, our approach enhances relevance to human health and may reveal pathway biases not apparent in single-compound studies.

PFAS are persistent, bioaccumulative compounds with long serum elimination half-lives (Buck et al. 2011; Sunderland et al. 2019; Cousins et al. 2020). Given that bioaccumulation of PFAS in the placenta has been demonstrated (Mamsen et al. 2019), it is possible that PFAS concentrations at the maternal–fetal interface are higher than those in peripheral blood. In an acute *in vitro* system, higher concentrations may therefore approximate the cumulative cellular stress associated with long-term PFAS burden during pregnancy, rather than transient systemic exposure. This framework supports the physiological relevance of the exposure range used to identify dominant stress-signaling pathways in STB cells. In addition, although the higher concentrations used here exceed population medians, they are within the range reported for occupationally exposed individuals (Woskie et al. 2012; Zhou et al. 2014; He et al. 2025), further supporting the physiological relevance of the higher concentrations in this study.

These findings have implications for understanding placental vulnerability to environmental toxicants. Excessive trophoblast stress and apoptosis are characteristic features of placenta-mediated pregnancy complications, including preeclampsia and fetal growth restriction (Sharp et al. 2010; Redman et al. 2022). Although the present study does not establish causal links between PFAS exposure and adverse pregnancy outcomes, identification of dominant stress-death pathways in STB cells provides mechanistic support for epidemiologic associations between prenatal PFAS exposure and placental pathology. Disruption of STB integrity through stress-induced apoptosis could plausibly impair placental transport and endocrine functions, thereby contributing to adverse developmental environments.

Several limitations should be acknowledged. PFAS exposures were acute and conducted in an *in vitro* system that does not capture maternal–fetal interactions, immune signaling, or chronic exposure dynamics. Nevertheless, the use of human trophoblast stem cell-derived STB, defined differentiation conditions, and pathway-specific pharmacologic inhibitors provides a powerful platform for mechanistic toxicology studies of placental cells.

Future studies should examine the effects of chronic or repeated low-dose PFAS exposure on placental stress signaling and determine whether prolonged activation of ISR and JNK pathways leads to adaptive remodeling or cumulative injury. Extension of these findings to additional placental cell types and more complex model systems will be important for defining how PFAS exposure perturbs placental function across gestation.

In summary, our data demonstrate that PFAS mixtures activate hierarchically organized stress signaling pathways in human STB cells, with JNK functioning as the dominant mediator of apoptosis and the ISR contributing in a secondary, modulatory capacity. These findings advance mechanistic understanding of PFAS-induced placental toxicity and highlight JNK signaling as a critical pathway linking PFAS exposure to placental cell injury.

## Data Availability

Raw data and materials generated by this study are available from the corresponding authors upon written request. Requests outlining the intended use of the data for academic research will be evaluated for compliance with institutional policies and applicable ethical requirements.

## Study approval

Experiments were approved by the Children’s Mercy Institutional Biosafety Committee (IBC00091).
