# Correction to: Stress-activated pathways mediate PFAS effects on human placental syncytiotrophoblast cells

**DOI:** 10.1093/toxsci/kfag117

**Published:** 2026-09-26

This is a correction to: Keisuke Kozai, Kaela M Varberg, Stress-activated pathways mediate PFAS effects on human placental syncytiotrophoblast cells, *Toxicological Sciences*, Volume 209, Issue 6, June 2026, kfag060, https://doi.org/10.1093/toxsci/kfag060

In the originally published version of this manuscript, Panels A and B are missing from Figure 3. It was erroneously cropped to only include Panel C.

This error has been corrected, and the figure has been updated with all panels.

